# Improvement of Image Sticking in Liquid Crystal Display Doped with γ-Fe_2_O_3_ Nanoparticles

**DOI:** 10.3390/nano8010005

**Published:** 2017-12-24

**Authors:** Wenjiang Ye, Rui Yuan, Yayu Dai, Lin Gao, Ze Pang, Jiliang Zhu, Xiangshen Meng, Zhenghong He, Jian Li, Minglei Cai, Xiaoyan Wang, Hongyu Xing

**Affiliations:** 1 School of Sciences, Hebei University of Technology, Tianjin 300401, China; wenjiang_ye@hebut.edu.cn (W.Y.); rui_yuanedu@163.com (R.Y.); 15102225686@163.com (Y.D.); 15983332112@163.com (L.G.); pangze111@163.com (Z.P.); zjl-656969@163.com (J.Z.); 2 School of Physical Science and Technology, Southwest University, Chongqing 400715, China; 15552267851@163.com (X.M.); hezhenho@swu.edu.cn (Z.H.); aizhong@swu.edu.cn (J.L.); 3 Hebei Jiya Electronics Co. Ltd., Shijiazhuang 050071, China; 4 Hebei Provincial Research Center of LCD Engineering Technology, Shijiazhuang 050071, China; cml@jiyalcd.com (M.C.); wwxxy@jiyalcd.com (X.W.)

**Keywords:** image sticking, dielectric property, liquid crystal, γ-Fe_2_O_3_ nanoparticles, capacitance model, dynamic response, threshold voltage

## Abstract

Image sticking in thin film transistor-liquid crystal displays (TFT-LCD) is related to the dielectric property of liquid crystal (LC) material. Low threshold value TFT LC materials have a weak stability and the free ions in them will be increased because of their own decomposition. In this study, the property of TFT LC material MAT-09-1284 doped with γ-Fe_2_O_3_ nanoparticles was investigated. The capacitances of parallel-aligned nematic LC cells and vertically aligned nematic LC cells with different doping concentrations were measured at different temperatures and frequencies. The dielectric constants perpendicular and parallel to long axis of the LC molecules ε_⊥_ and ε_//_, as well as the dielectric anisotropy Δε, were obtained. The dynamic responses and the direct current threshold voltages in parallel-aligned nematic LC cells for different doping concentrations were also measured. Although the dielectric anisotropy Δε decreased gradually with increasing temperature and frequency at the certain frequency and temperature in LC state for each concentration, the doping concentration of γ-Fe_2_O_3_ nanoparticles less than or equal to 0.145 wt % should be selected for maintaining dynamic response and decreasing free ions. This study has some guiding significance for improving the image sticking in TFT-LCD.

## 1. Introduction

The application of liquid crystal (LC) materials has widely permeated the display and non-display fields. LC display is realized by controlling the molecular orientation of LCs under an external voltage [1]. In the non-display field, LCs can modulate the phase through LC devices. The adjustment effect is improved by using different device structures and material designs, such as an in-plane switching mode [2], fringe field switching mode [3,4,5], multi-domain structure [6,7,8], polymer-stabilized LC [9,10,11,12,13], and polymer network LC [14,15,16]. Another method is doping nanoparticles into LC materials [17,18,19,20,21,22,23,24,25,26,27,28]. Nanoparticles doped into LC materials [26,27,28] can increase the dielectric anisotropy of them to decrease the threshold voltage. However, the low threshold value LC materials are unsuitable for thin film transistor-liquid crystal displays (TFT-LCD) because image sticking always occurs [29]. Image sticking plays an important role in the image quality of liquid crystal displays (LCDs), by which the previous pattern is still visible when the next pattern is addressed. Some papers have studied the improvement of the image sticking through the structure and the composition of TFT-LCD alignment film materials [30,31,32,33]. To reduce image sticking, reducing the transient bound ions on the alignment layers in LC materials is a feasible method. Low threshold value TFT LC materials have weak stability and the free ions in them will be increased because of their own decomposition, which leads to the increase of transient bound ions. So high threshold value TFT LC materials are usually chosen, such as MAT-09-1284 (Merck, Darmstadt, Germany). As a type of magnetic nanoparticle, γ-Fe_2_O_3_ nanoparticles doped into LC materials can also change the LC molecular orientation and thereby cause a magneto-optic effect [34]. On the other hand, the dielectric anisotropy of the liquid crystal material has a certain influence on the image sticking. Therefore, the dielectric properties are subject to further investigation. In this study, we investigated the dielectric property of LC material MAT-09-1284 doped with γ-Fe_2_O_3_ nanoparticles to explore how to improve image sticking in TFT-LCD.

The dielectric property of LC material mainly refers to the change of dielectric constants. In general, the factors that affect the LC dielectric constants include the LC molecular structure, LC blending, LC doping, temperature, and external voltage frequency. Once the LC composition is determined, only temperature and the external voltage frequency affect the dielectric constants of LCs. Given the electric anisotropy of the dielectric material, the dielectric constants of LC materials are described by ε_⊥_ and ε_//_, which represent perpendicular and parallel orientations, respectively, relative to the long axis of LC molecules. Consequently, the dielectric property of LC material MAT-09-1284 doped with γ-Fe_2_O_3_ nanoparticles can be investigated by measuring their dielectric constants.

Normally, the dielectric constants ε_⊥_ and ε_//_ can be obtained by measuring the capacitance of parallel-aligned nematic (PAN) cell under low voltage (less than the threshold voltage) and high voltage (greater than the saturation voltage) [35], respectively. However, great errors are highly likely under this method. First, the alignment layer on the glass substrate surface influences the measured capacitance of the LC cell, and its capacitance should also be considered [36]. Second, given the strong anchoring of the alignment layer to LC molecules in a PAN cell, guaranteeing the orientation of all LC molecules along the electric field is highly difficult even when a high voltage is applied to LC cells. Thus, measuring the dielectric constant ε_//_ as mentioned above causes an important problem. In this regard, the LC cell capacitance can be transformed into the LC layer capacitance by using the LC cell capacitance model. The dielectric constants ε_⊥_ and ε_//_ can be measured by the dual-cell method [37], namely, they are obtained by the LC layer capacitance of PAN cell for low voltage (less than the threshold voltage) and vertically aligned nematic (VAN) cell for high voltage, respectively.

Besides the dielectric property, the doped nanoparticles also affect other physical properties of the LC host, e.g., viscosity. For this purpose, the dynamic responses of LC material MAT-09-1284 doped with γ-Fe_2_O_3_ nanoparticles in PAN cells for different concentrations were also conducted and analyzed.

## 2. Preparation of LC Material and LC cell

### 2.1. Preparation of LC Material Doped with Nanomaterials

γ-Fe_2_O_3_ nanoparticles were prepared by the chemically induced transition method [38,39,40]. First, precursor FeOOH/Mg(OH)_2_ was synthesized by the chemical co-precipitation method. Subsequently, the resultant hydroxide precursor FeOOH/Mg(OH)_2_ was treated in the liquid phase with FeCl_2_ solution. During treatment, the Mg(OH)_2_ compound dissolved and the FeOOH dehydrated and transformed into γ-Fe_2_O_3_ nanoparticles. LC material MAT-09-1284 was purchased from Merck. It was a mixture of various LC monomers. The clearing point of the pure LC mixture without doping the γ-Fe_2_O_3_ nanoparticles was 80.5 °C, the perpendicular and parallel dielectric constants ε_⊥_ and ε_//_ at the temperature 25 °C are 5.2 and 2.6, respectively, and the dielectric anisotropy Δε at the temperature 25 °C is 2.6. γ-Fe_2_O_3_ nanoparticle is an inorganic material, and LC material MAT-09-1284 is an organic material; thus, the components are not mutually soluble. Only by coating γ-Fe_2_O_3_ nanoparticles with oleinic acid can they blend well together at different concentrations.

### 2.2. Preparation of LC Cell

**Figure 1:**
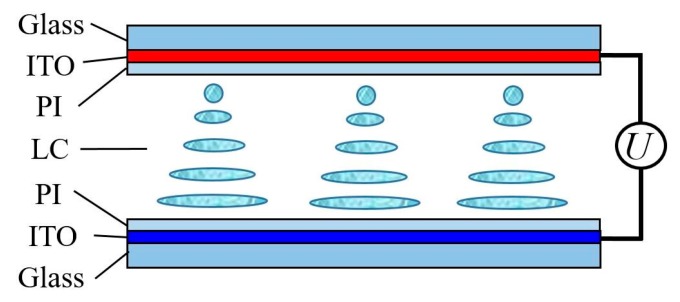
Schematic of LC cell capacitance model.

The schematic of a LC cell is shown in Figure 1. The LC cell comprises two etched indium tin oxide (ITO) glass substrates, each of which is coated with polyimide (PI) and sealed by a border adhesive. The LC cells used in this experiment include PAN and VAN cells, which were prepared with the following steps: ITO glass cleaning → drying → etching → cleaning → drying → PI coating → drying → rubbing → cleaning → drying → border sealing. As the alignment layer, the PI layer can guarantee the LC molecules homogeneous alignment, which is crucial to LC devices. The relative dielectric constants of the parallel and vertical PIs are all 3.1, and the pre-tilt angles on these two glass substrates in PAN and VAN cells are 1° and 89°, respectively. The effective electric field is a 1-cm-diameter circle which was etched in the ITO glass substrate.

### 2.3. LC Cell Capacitance Model

A LC cell can be viewed as a capacitor from its configuration (Figure 1). The cell possesses a capacitance composed of three parts, namely, the upper and lower PI alignment layer capacitances and the LC layer capacitance [36,41]. LC cell capacitor is equivalent to a series of three-layer capacitors. The measured capacitance actually corresponds to the capacitance of LC cell, and the external voltage is also applied to LC cell. However, the LC layer capacitance determines the dielectric constants of LC material. This observation indicates that LC cell capacitance must be transformed into LC layer capacitance by using the LC cell capacitance model.

LC cell capacitance *C* is given by

(1) $$\frac{1}{C}=\frac{1}{C_{1}}+\frac{1}{C_{\mathrm{LC}}}+\frac{1}{C_{2}}$$

where *C*_1_ = *S*ε_0_ε_1_/*L*_1_ and *C*_2_ = *S*ε_0_ε_2_/*L*_2_ correspond to the capacitances of upper and lower PI layers, respectively; *C*_LC_ is the capacitance of LC layer; *S* is the electrode area; ε_1_ and *L*_1_ are the relative dielectric constant and the thickness of the upper PI layer, respectively; and ε_2_ and *L*_2_ are the relative dielectric constant and the thickness of the lower PI layer, respectively. The expression of *C*_LC_ can be easily derived as

(2) $$C_{\mathrm{LC}}=\frac{CC_{1}C_{2}}{C_{1}C_{2}-CC_{2}-CC_{1}}$$

The external voltage *U*_LC_ applied to the LC layer is expressed as

(3) $$U_{\mathrm{LC}}=\frac{CU}{C_{\mathrm{LC}}}$$

where *U* is the external voltage applied to LC cell.

If the influence of the pre-tilt angles *θ*_PAN_ and *θ*_VAN_ on the substrate surface in PAN and VAN cells is considered, then the dielectric constants ε_⊥_ and ε_//_ of LC material satisfy the equation

(4) $$\left\{ \begin{array}{l} \frac{1}{C_{\mathrm{LC}-\mathrm{PAN}}}=\frac{L_{\mathrm{LC}-\mathrm{PAN}}}{S\varepsilon_{0}\left(\varepsilon_{⊥}+\Delta \varepsilon \sin^{2}\theta_{\mathrm{PAN}}\right)}\\ \frac{1}{C_{\mathrm{LC}-\mathrm{VAN}}}=\frac{L_{\mathrm{LC}-\mathrm{VAN}}}{S\varepsilon_{0}\left(\varepsilon_{⊥}+\Delta \varepsilon \sin^{2}\theta_{\mathrm{VAN}}\right)} \end{array}\right.$$

where *L*_LC-PAN_ and *L*_LC-VAN_ are the thicknesses of LC layer in PAN and VAN cells, respectively; Δε = ε_//_ − ε_⊥_ is the dielectric anisotropy of LC material; and ε_0_ is the vacuum dielectric constant.

## 3. Experiment

**Figure 2:**
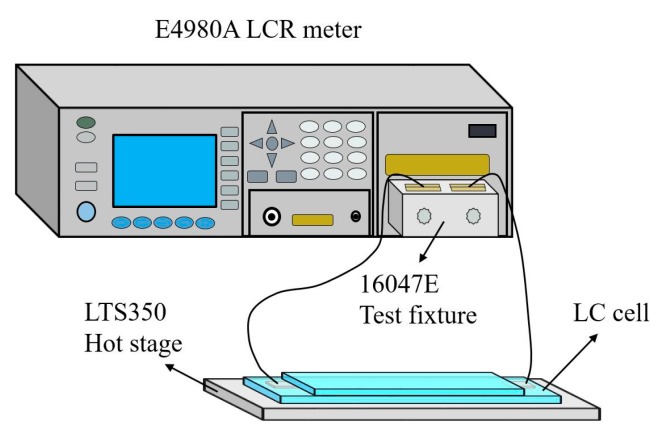
Experimental configuration for measuring the LC cell capacitance.

The instrument used to measure the LC cell capacitance was the precision LCR meter E4980A (Agilent, Palo Alto, CA, USA). The experimental configuration was shown in Figure 2A. Certain temperature was ensured in the measurement by mounting the LC cell on a hot stage LTS350 (Linkam, Surrey, UK) regulated by a hot controller TP94 (Linkam). At the same time, the effects of the lead wires and the alligator clips connected to the test fixture were eliminated by minimizing the length of the lead wires.

First, the LC material MAT-09-1284 doped with γ-Fe_2_O_3_ nanoparticles was used to fill the PAN and VAN cells, which were sealed by the ultraviolet (UV) sealing adhesive. During the UV curing process, the polarizer was attached on the glass substrate above the LC layer to protect the LC from UV light. Next, metal pins were added to both LC cell substrates through conductive adhesive, and the LC cell was fixed on a hot stage by high-temperature-resistant adhesive tape. Then, the test fixture 16047E (Agilent, Palo Alto, CA, USA) was connected to the precision LCR meter E4980A (Agilent). After setting up the measurement conditions of the precision LCR meter, the open/short correction function was applied to acquire further precise data. Finally, the capacitance data with different doping concentrations were recorded at different temperatures and frequencies under external voltages from 0.1 to 20 V and used to plot capacitance-voltage curves. Through the LC cell capacitance model, the dielectric constants and dielectric anisotropy could be obtained.

The LC layer capacitances of PAN and VAN cells under different voltages were obtained by accurately measuring the thicknesses of the LC layer (cell gap) and those of the upper and lower PI alignment layers. Through the double-beam UV and visible spectrophotometer UV-9000S (Metash, Shanghai, China), the average values of the cell gap of PAN and VAN cells were 3.95 and 4.00 μm, respectively. Given different PIs used in manufacturing PAN and VAN cells, the thicknesses of these two PI alignment layers differed. With the aid of the non-contact surface profilometer Contor GT-K (Bruker, Karlsruhe, Germany), the average thicknesses of the PI layers in PAN and VAN cells were 50 and 15 nm, respectively.

### 3.1. Influence of Temperature on the Dielectric Property

**Figure 3:**
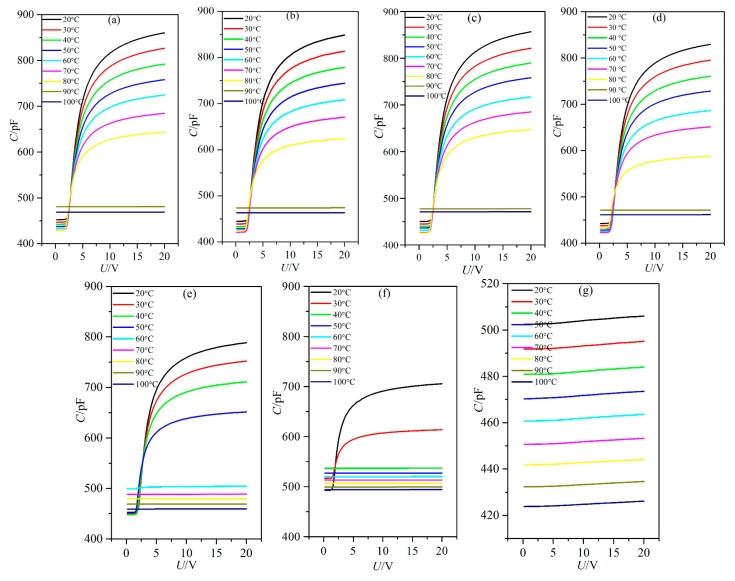
Liquid crystal (LC) cell capacitance versus voltage with frequency of 1 kHz for parallel-aligned nematic (PAN) cell under different temperatures and doped γ-Fe_2_O_3_ nanoparticle concentrations of (**a**) 0.0; (**b**) 0.02; (**c**) 0.048; (**d**) 0.145; (**e**) 0.515; (**f**) 0.984; and (**g**) 2.6 wt %.

**Figure 4:**
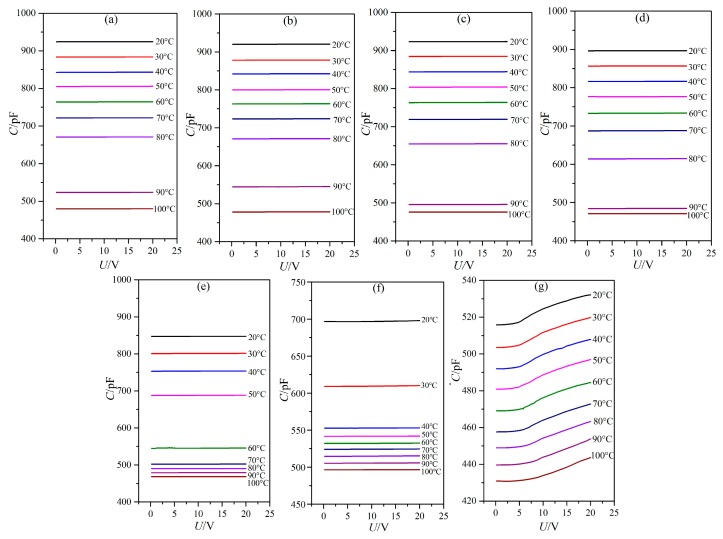
LC cell capacitance versus voltage with frequency of 1 kHz for vertically aligned nematic (VAN) cell under different temperatures and doped γ-Fe_2_O_3_ nanoparticle concentrations of (**a**) 0.0; (**b**) 0.02; (**c**) 0.048; (**d**) 0.145; (**e**) 0.515; (**f**) 0.984; and (**g**) 2.6 wt %.

The dielectric constants of LC materials were all known to be obviously influenced by temperature. Only when the temperature was within a certain range would the LC materials be in the LC state. The influence of temperature on the dielectric constants of the LC material MAT-09-1284 doped with γ-Fe_2_O_3_ nanoparticles was investigated under different concentrations. We measured the capacitances of PAN and VAN cells from the temperature of 20 to 100 °C by adjusting the hot controller, as shown in Figure 3 and Figure 4. The concentrations of doped γ-Fe_2_O_3_ nanoparticles were (a) 0.0; (b) 0.02; (c) 0.048; (d) 0.145; (e) 0.515; (f) 0.984; and (g) 2.6 wt %. The frequency of the external voltage was 1 kHz.

**Figure 5:**
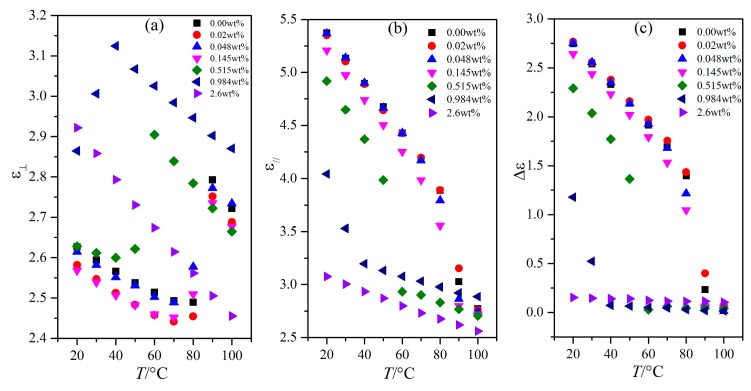
Dependence of the dielectric constants (**a**) ε_⊥_ and (**b**) ε_//_ and the dielectric anisotropy (**c**) Δε on temperature for the LC material MAT-09-1284 doped with γ-Fe_2_O_3_ nanoparticles of different concentrations.

From the microscopic viewpoint, the dielectric constants ε_⊥_ and ε_//_ of the LC material MAT-09-1284 doped with γ-Fe_2_O_3_ nanoparticles are related to the molecular polarization, the order parameter, the angle between the permanent dipole moment and the molecular long axis, and the magnetization of γ-Fe_2_O_3_ nanoparticles excited by the electric field, etc. This is a complicated change which can be reflected by the capacitance of LC cell. When the temperature was certain and the voltage was lower than the threshold voltage, the capacitance of PAN cell was only related to the molecular polarization and the magnetization. The general tendency of the capacitance was initially decreased and then increased before the temperature reached the clearing point as the nanoparticle concentration increased, as shown in Figure 5a. The clearing point decreased with the increase in the concentration of doped γ-Fe_2_O_3_ nanoparticles (Figure 3). When the doping concentration reached 2.6 wt %, the capacitance achieved a small change, which indicated that the doped γ-Fe_2_O_3_ nanoparticles induced the LC material MAT-09-1284 to assume an almost isotropic state. This result revealed that γ-Fe_2_O_3_ nanoparticles doped into LC materials changed the LC molecular orientation by their magnetizations. In VAN cells, the capacitance decreased monotonously and remained unchanged with different voltages for the doping concentrations less than 2.6 wt %, under which around a dozen changed in the capacitance (Figure 4). The corresponding changes in the dielectric constant ε_//_ were shown in Figure 5b. This result illustrated that the LC material MAT-09-1284 doped with γ-Fe_2_O_3_ nanoparticles remained in the LC state, and the dielectric anisotropy Δε decreased with the increase in temperature. When the doping concentration was less than or equal to 0.145 wt % and the temperature was below the clearing point, the dielectric anisotropy changed slightly, especially at 0.048 wt % (Figure 5c). This result revealed that such concentration did not affect the dielectric anisotropy Δε of the LC MAT-09-1284 even though the dielectric constants ε_⊥_ and ε_//_ have some differences.

### 3.2. Influence of External Voltage Frequency on the Dielectric Property

**Figure 6:**
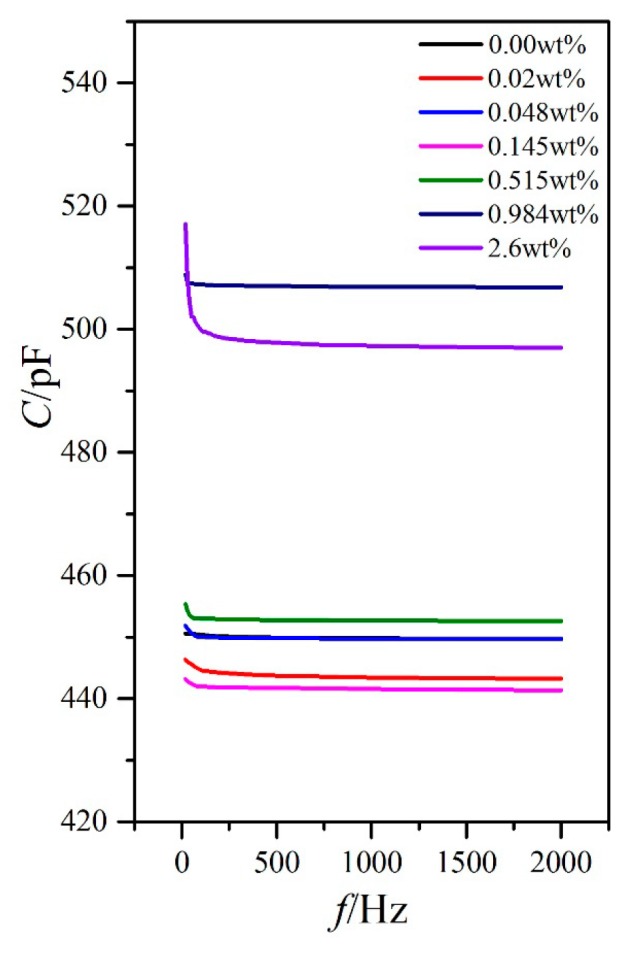
PAN cell capacitance versus frequency with different doped γ-Fe_2_O_3_ nanoparticle concentrations under the external voltage of 1 V and temperature of 25 °C.

**Figure 7:**
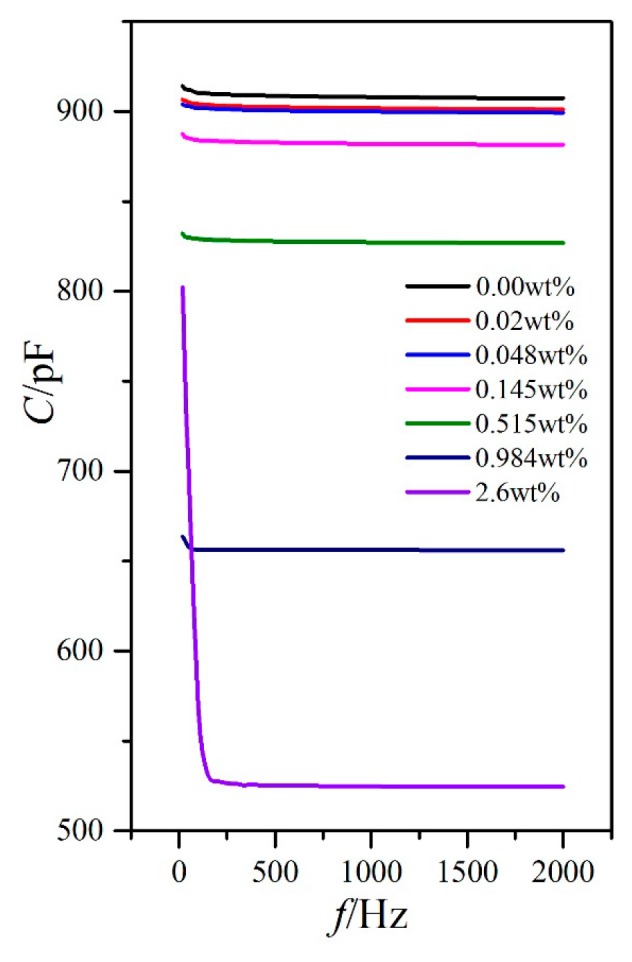
VAN cell capacitance versus frequency with different doped γ-Fe_2_O_3_ nanoparticle concentrations under the external voltage of 15 V and temperature 25 °C.

Aside from temperature, the external voltage frequency can also affect the LC dielectric constants. The temperature of the hot stage was stabilized to 25 °C, and the external voltage frequency was then changed by adjusting the precision LCR meter from 20 Hz to 2 kHz. The capacitances of PAN cell with an external voltage of 1 V (less than the threshold voltage in Figure 3) and VAN cell with an external voltage of 15 V (aligned well along the direction of the electric field) for the LC material MAT-09-1284 doped with γ-Fe_2_O_3_ nanoparticles of different concentrations were measured (Figure 6 and Figure 7).

**Figure 8:**
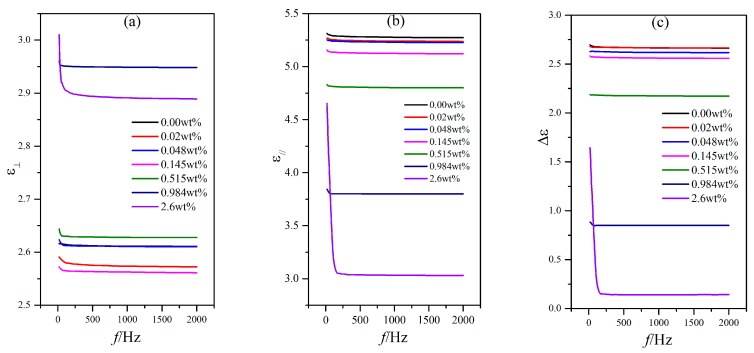
Dependence of the dielectric constants (**a**) ε_⊥_; (**b**) ε_//_ and the dielectric anisotropy (**c**) Δε on the frequency for the LC material MAT-09-1284 doped with γ-Fe_2_O_3_ nanoparticles of different concentrations.

For PAN and VAN cells, the capacitance variations with the external voltage frequency for different doping concentrations were extremely small (only several pF) within the frequency measurement range, except for the relatively low frequency of less than 100 Hz (Figure 6 and Figure 7). This result inevitably resulted in the small variations of the dielectric constants ε_⊥_ and ε_//_ and the dielectric anisotropy Δε to the frequency, as shown in Figure 8. In these cases, the changes of the dielectric constants were controlled by molecular polarization and magnetization. Although these variation rules on the dielectric constants ε_⊥_ and ε_//_ had some differences, the dielectric anisotropy Δε was decreased gradually with increased doping concentration for a certain frequency. When the doping concentration was less than 0.145 wt %, the difference was relatively small.

### 3.3. Dynamic Response and DC Threshold Voltage

Through the preceding analyses on the dielectric property of LC material MAT-09-1284 doped with γ-Fe_2_O_3_ nanoparticles, an appropriate doping concentration could be selected to decrease the dielectric anisotropy for improving the image sticking. However, the response time for such doping concentration should be guaranteed. Meanwhile, to explain the cause of improving the image sticking in nature, the behaviors of LC material MAT-09-1284 with different doping concentrations under DC drive should also be analyzed.

**Figure 9:**
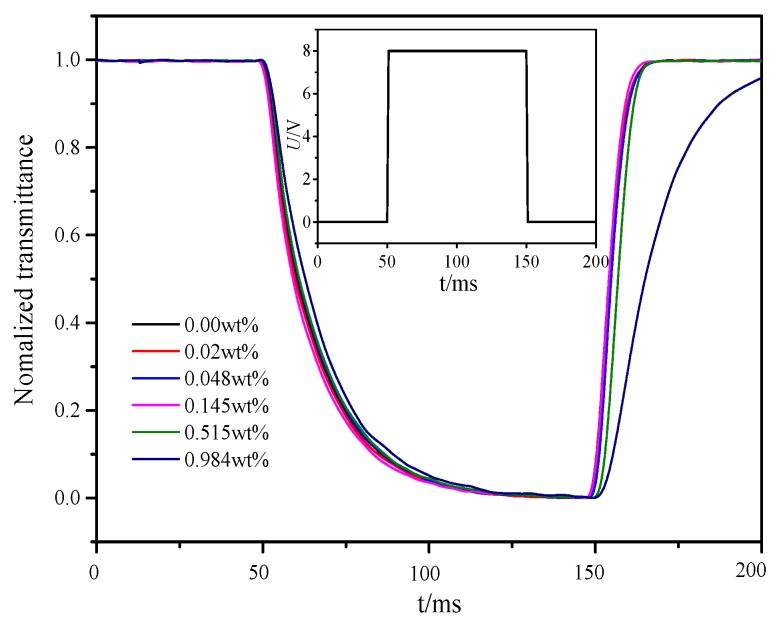
Normalized transmittance versus time of the LC material MAT-09-1284 doped with γ-Fe_2_O_3_ nanoparticles for different concentrations in PAN cell. The embedded diagram describes the characteristic of the pulse voltage. The pulse width is 100 ms and the amplitude is 8 V.

**Figure 10:**
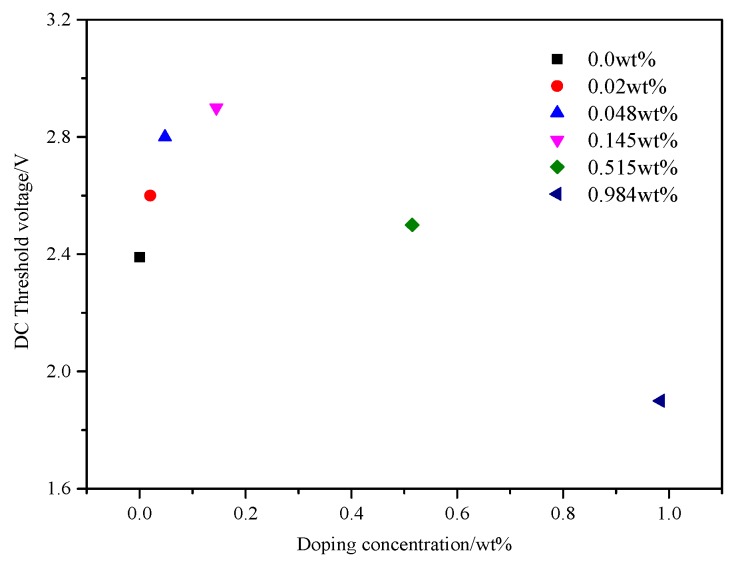
DC threshold voltage versus doping concentration of the LC material MAT-09-1284 doped with γ-Fe_2_O_3_ nanoparticles in PAN cell.

Impulse voltage with time 100 ms and amplitude 8 V or a DC voltage from 0 to 20 V was applied to PAN cells. The changes in the intensity of a laser beam (632.8 nm) that passed through the PAN cells were monitored by a detector connected to the oscilloscope (Tektronix MDO3024, Johnston, OH, USA). The temperature was controlled at 20 °C. The curves of normalized transmittance versus time and DC threshold voltage versus doping concentration in PAN cells were shown in Figure 9 and Figure 10, respectively.

The dynamic responses of LC material MAT-09-1284 for different doping concentrations less than or equal to 0.145 wt % were almost identical which could be seen from Figure 9. In addition, it was inevitable that there would be ions in the LC materials, as well as in the LC MAT-09-1284. These ions in PAN cells could be divided into two parts: free ions moving with the electric field and transient bound ions on the alignment layers. When the voltage induced by the bound ions was greater than the threshold voltage, image sticking would occur [42]. The free ions, however, could counteract the ion electric field to reduce the occurrence of image sticking. When a small amount of γ-Fe_2_O_3_ nanoparticles (≤0.145 wt %) was doped into the LC material MAT-09-1284, the magnetization of nanoparticles excited by electric field might adsorb the free ions, which would result in the increase of the DC threshold voltage, as shown in Figure 10. In this case, the magnetization was ordered. As increasing the doping concentration, the order of magnetization decreased and more free ions moved to the alignment layers to reduce the DC threshold voltage. Therefore, the dynamic response and the DC threshold voltage considered, the doping concentration (≤0.145 wt %) should be selected to improve the image sticking.

### 3.4. Long Term Stability of the LC Mixture

**Figure 11:**
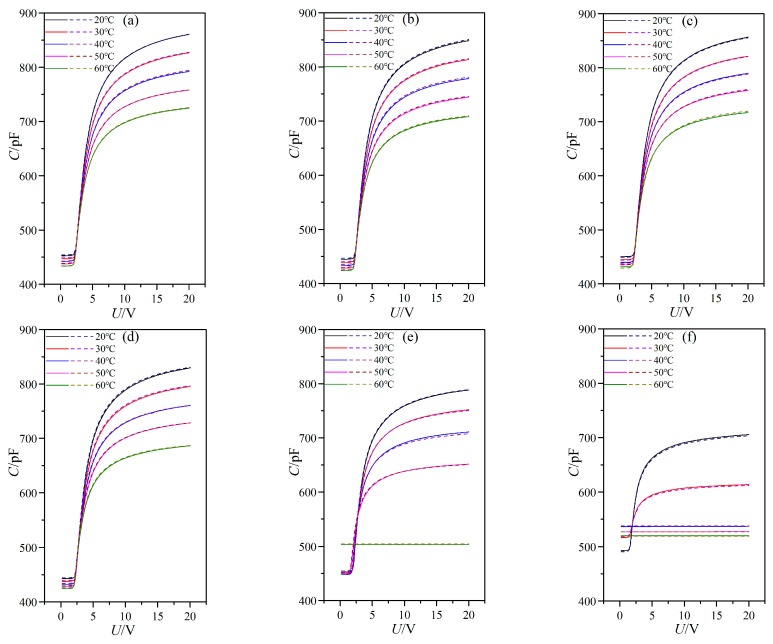
LC cell capacitance versus voltage with frequency of 1 kHz for PAN cell under different temperatures and doped γ-Fe_2_O_3_ nanoparticle concentrations of (**a**) 0.0 wt %; (**b**) 0.02 wt %; (**c**) 0.048 wt %; (**d**) 0.145 wt %; (**e**) 0.515 wt %; and (**f**) 0.984 wt %. Solid lines for the original data and dashed lines for the data a month later.

Most nanoparticle suspended LC systems suffer from long term stability issues. For investigating the long term stability of LC material MAT-09-1284 doped with γ-Fe_2_O_3_ nanoparticles of different doping concentrations, we controlled the same experimental environment as before and remeasured the capacitance of PAN cells at different temperatures and the frequency of 1 kHz to compare them with the original data from one month prior, as shown in Figure 11. The concentrations of doped γ-Fe_2_O_3_ nanoparticles were (a) 0.0 wt %; (b) 0.02 wt %; (c) 0.048 wt %; (d) 0.145 wt %; (e) 0.515 wt %; and (f) 0.984 wt %.

These curves of capacitance versus voltage were coincident for two independent measurements. It showed that γ-Fe_2_O_3_ nanoparticles used in experiment blended well with the LC material MAT-09-1284 and they did not aggregate in the LC host as the time increased. In other words, the LC material MAT-09-1284 doped with γ-Fe_2_O_3_ nanoparticles of different doping concentrations possessed long term stability.

## 4. Conclusions

In this study, through investigating the effect of temperature and frequency on the dielectric property of LC material MAT-09-1284 doped with γ-Fe_2_O_3_ nanoparticles of different doping concentrations, the dynamic response and the DC threshold voltage in PAN cell, an improvement of image sticking in TFT-LCD was proposed. In addition to selecting the high threshold value TFT LC materials, doping γ-Fe_2_O_3_ nanoparticles into the LC materials could also improve the image sticking because of their magnetization induced by the electric field. Compared to the pure LC material MAT-09-1284, a doping concentration less than or equal to 0.145 wt % of γ-Fe_2_O_3_ nanoparticles could be chosen. The best doping concentration was 0.145 wt %, with which the long term stability of LC mixture could be maintained. This study has some guiding significance for improving image sticking in TFT-LCD.
